# LDL acts as an opsonin enhancing the phagocytosis of group A *Streptococcus* by monocyte and whole human blood

**DOI:** 10.1007/s00430-015-0436-8

**Published:** 2015-09-21

**Authors:** Lulei Zhou, Ling Liu, Jinli Yang, Yuxin Li, Wencheng Bai, Na Liu, Wenlong Li, Yumin Gao, Liping Xu, Zhi Liu, Runlin Han

**Affiliations:** Research Center of Plasma Lipoprotein Immunology, Inner Mongolia Agricultural University, Huhhot, China; Key Laboratory of Animal Clinic Diagnosis and Treatment, Ministry of Agriculture of China, Inner Mongolia Agricultural University, Huhhot, China; College of Public Health, Inner Mongolia Medical University, Huhhot, China; College of Basic Medicine, Inner Mongolia Medical University, Huhhot, China

**Keywords:** Low-density lipoprotein, Group A *Streptococcus*, Opsonin, Phagocytosis, Monocyte

## Abstract

Low-density lipoprotein (LDL) binds to group A *Streptococcus* (GAS) through Sc11 protein, and scavenger receptor CD36 of monocyte mediates the endocytosis of native or modified LDL. Therefore, we hypothesized that LDL might be an opsonin enhancing the phagocytosis of LDL-bound GAS by monocyte. The results showed that LDL could significantly promote U937 cell to phagocytose M28 (ATCC BAA1064) and M41 (ATCC 12373, AM41)-type GAS, and the phagocytosis rates were significantly increased, compared with LDL-free group. LDL, however, did not enhance the phagocytosis of M41 (CMCC 32198, CM41) or M6 (ATCC BAA946)-type GAS since these two strains did not bind to LDL. CD36 was the major scavenger receptor mediating the uptake of LDL-bound GAS by monocyte U937 cells since anti-CD36 antibody abolished the phagocytosis of LDL-opsonized GAS but anti-CD4 antibody did not. Most of AM41-type GAS cells were killed in human blood, whereas only a few CM41-type cells were phagocytosed. Moreover, recombinant Scl1 (rScl1) derived from M41-type GAS could significantly decrease the opsonophagocytosis of AM41 but not CM41-type GAS because the rScl1 competitively blocked the binding of AM41-type GAS to LDL. Therefore, our findings suggest that LDL may be an opsonin to enhance CD36-dependent opsonic phagocytosis of GAS by monocyte.

**Electronic supplementary material:**

The online version of this article (doi:10.1007/s00430-015-0436-8) contains supplementary material, which is available to authorized users.

## Introduction

*Streptococcus pyogenes* [group A *Streptococcus* (GAS)] can cause a number of suppurative infections, including pharyngitis, impetigo/pyoderma, erysipelas, cellulitis, necrotizing fasciitis, toxic streptococcal syndrome, and scarlet fever, as well as nonsuppurative sequelae including acute rheumatic fever and acute glomerulonephritis [1]. Based on the surface M protein, GAS is serologically separated into over 200 M protein serotypes [2]. Streptococcal collagen-like protein 1 (Scl1) as one of the virulence factors of GAS is found on the cell surface of many M-type GAS [3]. Although Scl1s expressed by different M-type GAS share similar triple helix structure their amino acid sequence, especially in variable N-terminal region, varies significantly, so that Scl1s in M6 and M55-type GAS specifically bind to factor H [4], whereas Scl1s in M1, M12, M28 and M41 can interact with low-density lipoprotein (LDL) [5]. However, the significance of Scl1–LDL interaction remains unknown.

HDL and LDL especially OxLDL were found to play anti-infectious role in protecting bacteria, viruses, or parasites infections in some studies, regardless of some contradicting findings in other studies [6]. Scavenger receptor CD36 mediates the uptake of modified and native LDL and HDL by monocyte [7, 8]; however, OxLDL upregulates CD36, whereas HDL downregulates CD36 via PPAR-mediated pathways [7].

Therefore, we hypothesize that LDL may be an opsonin to interact with Scl1 to enhance the phagocytosis of LDL-bound GAS by monocyte/macrophage.

## Materials and methods

### Bacterial cultures and fluorescence labeling

Three GAS strains M6 (ATCC BAA946), M28 (ATCC BAA1064), and M41 (ATCC 12373, AM41) were purchased from American Tissue Culture Collection, and one strain M41 (CMCC 32198, CM41) was obtained from China Medical Culture Collection Center, respectively. *Scl1* nucleotide sequence of CM41 is the same as that of AM41 (GenBank: EU915249.1). Some characteristics of four GAS strains are listed in Table 1. Scl1 expression and LDL binding capacity of the four GAS strains were assayed using RT-PCR and ELISA as described previously [5]. Based upon RT-PCR analysis, scl1 gene was expressed in AM41-type GAS but not in natural mutant CM41-type GAS (Figure S1A). Moreover, AM41-type GAS could bind to LDL but the interaction of CM41-type GAS with LDL was weak as demonstrated with ELISA (Figure S1B).

**Table 1:** Characteristics of GAS strains

GAS strain	*Scl1* expression	Binding to LDL
M41 (ATCC 12373, AM41)	+	+
M41 (CMCC 32198, CM41)	−	−
M28 (ATCC BAA1064)	+	+ [5]
M6 (ATCC BAA946)	+	− [5]

GAS cultures were grown on brain–heart infusion agar (BD Biosciences, USA) overnight and in Todd Hewitt broth (BD Biosciences, USA) supplemented with 0.2 % yeast extract (THY medium) at 37 °C in an atmosphere of 5 % CO_2_. Logarithmic phase cultures harvested at the optical density (600 nm) of about 0.5–0.6 (OD600 nm) were used to prepare GAS. Colony counts were verified by plating on THY agar.

Prior to use in uptake assays, bacteria were labeled with fluorescein isothiocyanate (FITC, Sigma, USA) using method described previously [9]. Briefly, 10 ml of exponentially growing bacterial culture was pelleted and washed twice with sterile phosphate-buffered saline (PBS). The bacterial pellet was resuspended in 1 ml labeling solution (0.2 mg/ml FITC in PBS) and incubated in an atmosphere of 5 % CO_2_ at 37 °C for 30 min. The FITC-labeled bacteria were pelleted, washed once with cold PBS, and resuspended in RPMI 1640 medium (Gibco, USA) at a final concentration of ~10^9^ cfu/ml.

### Recombinant protein

Recombinant Scl1 (rScl1, C176) derived from M41-type GAS was produced in *E. coli* using the *Strep*-tag II expression and purification system (IBA-GmbH, Germany) as described previously [5, 10]. Briefly, six milliliter overnight pre-cultures of *E. coli* BL21 was inoculated into 300 ml of Luria–Bertani (LB) broth containing 100 μg/ml ampicillin and incubated at 37 °C under agitation (200 rpm). When OD600 reached 0.5–0.6, protein expression was induced by addition of anhydrotetracycline (0.2 μg/ml) and incubated at 30 °C under agitation (200 rpm) for another 3 h. Bacteria were harvested by centrifugation (10,000×*g*, 10 min, 4 °C), and the pellets were resuspended in a lysis buffer (100 mM Tris–HCl, 1 mM EDTA pH 8.0, 500 mM sucrose, 0.2 M PMSF) containing 10 μl lysozyme and 20 μl DNase. After incubated at room temperature for 30 min, the lysates were clarified by centrifugation (10,000×*g*, 10 min, 4 °C), and the recombinant polypeptides in supernatant were purified by affinity chromatography with *Strep*-Tactin Sepharose.

The protein content in the elution was determined by BCA™ Protein Assay Kit (Pierce Biotechnology, USA) using bovine serum albumin as a standard. Endotoxin in rScl1 preparations was removed by high-capacity endotoxin removal resin (Pierce Biotechnology, USA). The endotoxin content of rScl1 solution was <3 EU/ml determined by LAL Chromogenic Endotoxin Quantitation Kit (Pierce Biotechnology, USA).

### Cell cultures

Most of the macrophage scavenger receptors, including class A, B, D, E, F and G, can mediate the internalization of LDL and/or oxLDL, whereas only class B receptors, including Type I, Type II and CD36, present on monocyte [7]. Therefore, human monocytic tumor cell line U937 was used as a simple model to explore LDL-mediated opsonophagocytosis in the current study. Human monocytic U937 cells (ATCC CRL-1593.2, USA) were maintained in RPMI1640 medium supplemented with 10 % heat-inactivated fetal calf serum (Gibco, Australia), 292.28 µg/ml l-glutamine and 100 units/ml penicillin**/**streptomycin (Gibco, USA). In the phagocytosis assays, cells were harvested and re-suspended in RPMI 1640 medium at a final concentration of 2.5 × 10^6^/ml.

### In vitro phagocytic assay

#### LDL-mediated phagocytic assay

For opsonophagocytosis experiments, 100 µl of FITC-labeled GAS (~10^9^ cfu/ml) with or without LDL (10 µg/ml final concentration of protein) (Biomedical Technologies, USA) was added into 1 ml of U937 cells (2.5 × 10^6^/ml) and incubated at 37 °C in an atmosphere of 5 % CO_2_. The final bacteria/monocytes ratio was about 15–50:1. The viable bacterium number in the mixture was determined by plating diluted samples onto THY agar plates after 15-, 30-, 60-min incubation. To monitor bacterial fluorescence intensity after the incubation, 200 µl of sample was pipetted into the 96-well microplate and measured at *λ* = 495 nm (excitation) and *λ* = 519 nm (emission) using a fluorescence plate reader (model Synergy HT, BioTek, USA).

To test the influence of LDL concentration on opsonophagocytosis, 10, 100 or 1000 µg/ml of LDL was used. To study the competitively inhibitory effect of rScl1 on the LDL-mediated phagocytosis, assays were performed in essentially the same manner as described above except LDL was pre-incubated with an equal mole (20 nmol) of rScl1 for 1 min before adding to the cell culture.

To determine whether scavenger receptor CD36 was involved in the LDL-mediated phagocytosis of GAS by monocyte U937, monoclonal anti-CD36 antibody (SMO, Ancell, USA) was added to the cell suspension (10 µg/ml final concentration) to block the receptor before the phagocytosis assay. Irrelevant monoclonal anti-CD4 antibody (Ancell, USA) was used as the negative control (10 µg/ml final concentration).

#### Microscopy

In above LDL-mediated phagocytic assay, a 100 µl aliquot of U937 cells and FITC-labeled GAS mixture was taken out after 30-min incubation and placed on ice. DAPI (Sigma, USA) was added at a final concentration of 10 µg/ml. After 10-min incubation, five milliliters of sample was plated onto slides, and FITC-GAS and DAPI-stained U937 cell nuclei were imaged at 200× magnification on fluorescent microscope (model BX41, Olympus, Japan) equipped with MicroPublisher 3.3 RTV camera (Qimaging, Canada). After acquisition, two photographs of GAS (green) and nuclei (blue) in the same field of view were merged using Image Pro Plus 6.0 software.

### Ex vivo phagocytic assay with human blood

Blood was obtained from healthy volunteers in accordance with Inner Mongolia Agricultural University institutional regulations. Whole blood was collected into 5-ml pyrogen-free vacuum tubes containing 3.2 % sodium citrate as anticoagulant. A 200 µl aliquot of blood was pre-incubated with 50 µl rScl1 or PBS at 37 °C under agitation (170 rpm) for 1 min. A 100 µl aliquot of AM41-type or CM41-type GAS (~10^3^ cfu/ml) was added into the blood and agitated for 15 min at 37 °C. The cfu was determined by plating samples on THY agar plates.

### Ethics statement

Experiments with human blood were approved by Academic Board and Science and Technology Department of the Inner Mongolia Agricultural University. The blood was taken from the volunteers with their authorization. All adult subjects signed written informed consent form, and children were not invited to participate in this project.

## Statistics

All treatments were performed in triplicate, and all experiments were conducted for three times. All data are presented as the mean ± SD. Statistical significance was performed using the two-tailed paired Student’s *t* test. Significance was determined at a level of [*p* < 0.001 (***)].

## Results

### LDL promotes the phagocytosis of GAS by human U937 monocyte

**Fig. 1:**
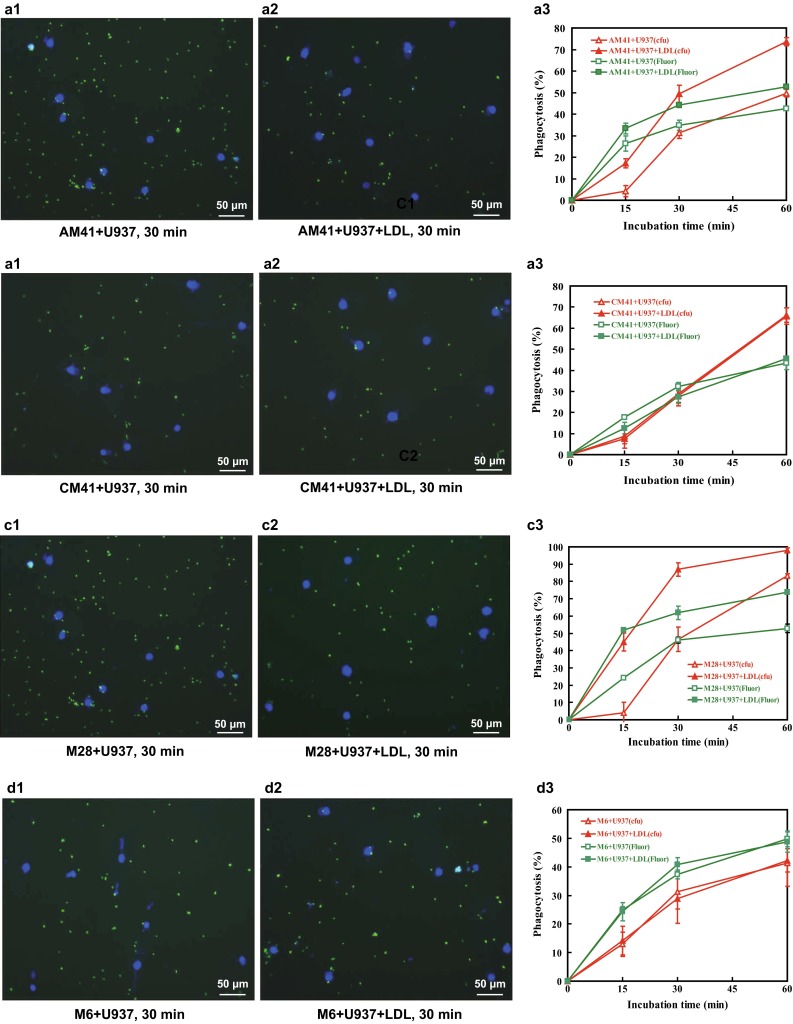
LDL-mediated phagocytosis of four GAS strains. After 30-min incubation of U937 cells with FITC-labeled GAS DAPI was added and co-cultivated for 10 min; 5 ml of sample was plated onto slides, and GAS (*green*) and DAPI-stained U937 cell nuclei (*blue*) were imaged, respectively, at ×200 magnification on fluorescent microscope. **a1**, **b1**, **c1** and **d1** refer to control group, including AM41, CM41, M28 and M6-type GAS without LDL, whereas **a2**, **b2**, **c2** and **d2** stand for the treatments with LDL. The colony-forming units (cfu) of GAS in the mixture were determined by plating diluted samples onto THY agar plates after 15-, 30- and 60-min co-culture of FITC-GAS and U937 cells in the presence or absence of LDL. The phagocytosis rates were expressed as 100 % × (cfu of addition − cfu after co-culture)/cfu of addition. The *red lines* in **a3**, **b3**, **c3** and **d3** refer to the phagocytosis rates calculated upon cfu. Bacterial fluorescence intensity was monitored at 15, 30 and 60 min of incubation, and 200 µl of sample was pipetted into the 96-well microplate and measured at *λ* = 495 nm (excitation) and *λ* = 519 nm (emission) using a fluorescence plate reader. The phagocytosis rates were expressed as 100 % × (initial absorbance value − absorbance value after co-culture)/initial absorbance value. The *green lines* in **a3**, **b3**, **c3** and **d3** refer to the phagocytosis rates calculated upon fluorescence (fluor)

To test whether LDL can promote the phagocytosis of GAS by monocyte, colony counting, fluorescence intensity detection and microscopy observation were employed (Fig. 1). Based upon colony counts, LDL significantly enhanced the phagocytosis of M41-type GAS (ATCC 12373, AM41-type GAS). The phagocytosis rates were increased by 17, 58 and 48 % at 15, 30 and 60 min, respectively, compared with LDL-free control (*p* < 0.01, Fig. 1a3). Similar results were obtained with fluorescence intensity detection. The uptake efficiency was increased by 17.6, 26.6 and 23.6 % at 15, 30 and 60 min, respectively, compared with LDL-free control (Fig. 1a3). The phagocytosis rates determined by fluorescence intensity were usually lower than those calculated with colony counts, because the intracellular FITC-labeled bacteria still fluoresced faintly. The opsonic phagocytosis was further demonstrated with microscopy (Fig. 1a1, LDL-free control; Fig. 1a2, LDL-positive group). In contrast to AM41-type GAS, the addition of LDL did not increase the phagocytosis rates of M41-type GAS (CMCC 32198, CM-41-type GAS) by U937 cells (Fig. 1b1–b3). The results also showed that LDL significantly promoted the ingestion of M28-type GAS (*p* < 0.001, Fig. 1c1–c3) but not M6-type GAS (Fig. 1d1–d3) since the former could bind to LDL but the latter could not. In overall, our data consistently demonstrated that LDL-opsonized GAS was phagocytosed more efficiently than GAS alone. To investigate whether internalized GAS was still alive 1 h after phagocytosis, U937 cells co-cultivated with GAS were lysed and plated onto THY agar plate [9, 11]. The colony-forming unit (cfu) of the total intracellular and extracellular live bacteria was not different from that of bacteria without the lysis of U937 cells (data not shown). These results indicated that GAS was dead after ingestion by U937 cells. In addition, LDL alone promoted the growth of all tested GAS strains in the absence of U937 cells (data no shown) because LDL may act as a nutrient substance for GAS.

### Mechanism underlying the LDL-mediated phagocytosis of GAS

#### Inhibition of LDL-mediated GAS phagocytosis by recombinant Scl1 (rScl1)

**Fig. 2:**
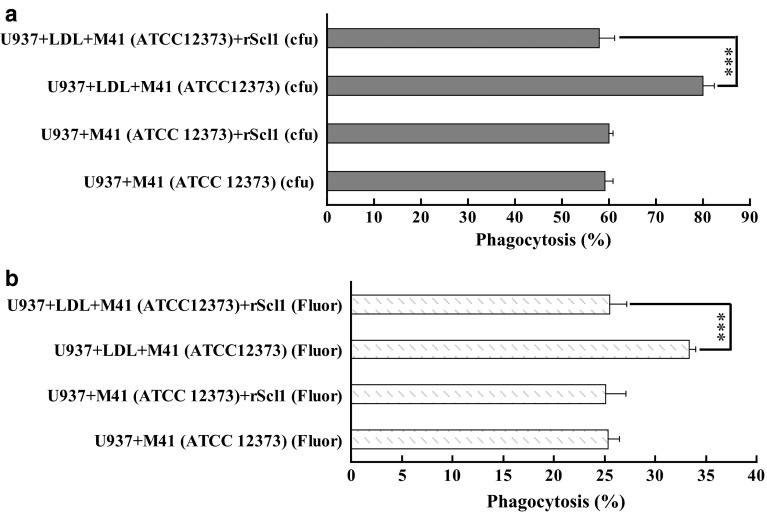
rScl1 inhibited LDL-mediated phagocytosis of GAS by U937 cells. The phagocytosis assay was carried out with similar procedure to Fig. 1 except the addition of rScl1. **a** The phagocytosis rates were calculated based upon cfu after 30-min incubation of U937 cells and GAS. **b** The phagocytosis rates were derived from fluorescence intensity (fluor) after 30-min co-culture of U937 cells and GAS

To test whether the binding of LDL to surface Scl1 of GAS was responsible for the opsonophagocytosis, rScl1 was added to the mixture of GAS, LDL and U937 cells since rScl1 could competitively inhibit the interaction of LDL with GAS (Figure S2). The results showed that rScl1 abolished the LDL-mediated opsonophagocytosis of AM41-type GAS but did not have any impact on LDL-free non-opsonic phagocytosis, which was consistent with the findings by fluorescence detection (Fig. 2a) and colony counting (Fig. 2b) after 30 min of co-cultivation of U937 cells with FITC-labeled GAS.

#### Anti-CD36 antibody abrogated the LDL-dependent phagocytosis of GAS by U937 cells

**Fig. 3:**
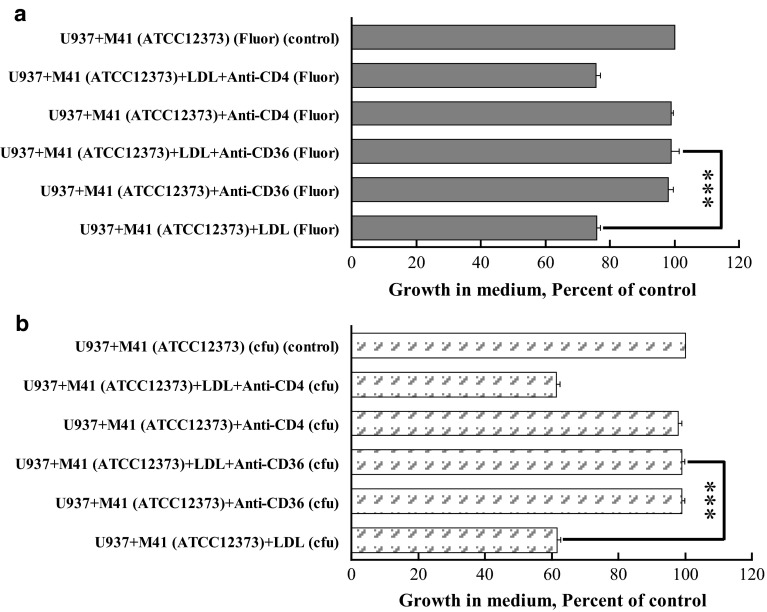
Role of anti-CD36 antibody in resistance to phagocytosis of GAS by U937 cells. The phagocytosis assay was carried out with similar procedure to Fig. 1 except the addition of anti-CD36 antibody or anti-CD4 antibody. Percent of control = (viable GAS in other treatments/viable GAS of U937 cells and GAS group) × 100 was determined after 30-min co-culture and calculated from cfu (**a**) and fluorescence intensity (**b**), respectively

To investigate which scavenger receptor of U937 cells was involved in LDL-mediated phagocytosis of GAS, monoclonal anti-CD36 antibody or an irrelevant anti-CD4 antibody was added into the culture. In the presence of anti-CD36 antibody, extracellular bacterial cfu and fluorescence intensity of U937 cells, LDL and GAS culture were increased to the same level as the control (Fig. 3a, b). In contrast, the addition of anti-CD4 antibody did not increase the extracellular bacterial growth (Fig. 3a, b). Therefore, anti-CD36 antibody abrogated the LDL-dependent phagocytosis of AM41-type GAS but did not decrease the LDL-independent phagocytosis. On the other hand, anti-CD4 antibody did not have any influence on either LDL-dependent or LDL-independent phagocytosis. These results indicated that CD36 was responsible for the recognization of LDL–GAS complex, leading to the rapid uptake of GAS by monocyte.

#### High concentration of LDL did not inhibit the LDL-mediated phagocytosis

**Fig. 4:**
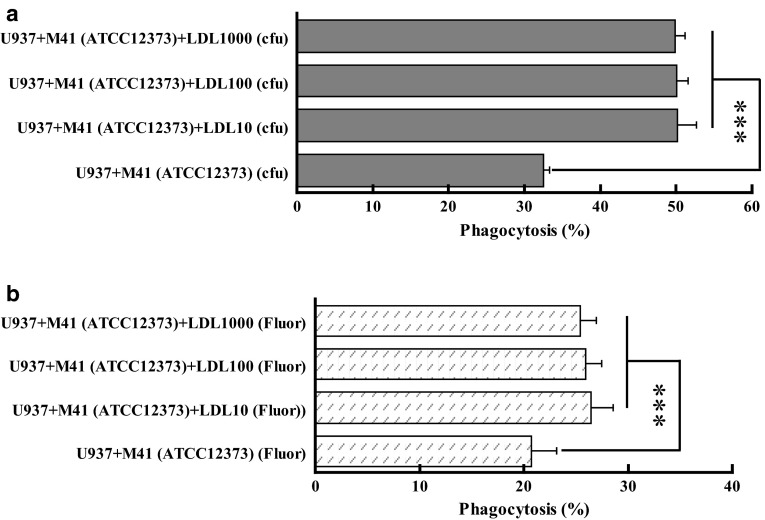
Effect of LDL concentration on the opsonic phagocytosis of GAS by U937 cells. The phagocytosis assay was carried out with similar procedure to Fig. 1 except the variation of LDL concentration from 10 to 1000 µg/ml. **a** The phagocytosis rates were calculated based upon cfu after 30-min incubation of U937 cells and GAS. **b** The phagocytosis rates were derived from fluorescence intensity (fluor) after 30-min co-culture of U937 cells and GAS

CD36 is a receptor of not only LDL [8] but also oxLDL [7]. Moreover, even freshly isolated LDL from human blood still contained some oxLDL [12]. In the above experiments, 10 μg/ml of LDL which is less than physiological concentration (about 1000 μg/ml) was used. Therefore, high concentration of LDL was tested; 100 or 1000 μg/ml of LDL was used to test whether LDL could competitively inhibit CD36 from recognizing LDL–GAS complex. The results showed that high concentration of LDL did not significantly decrease the phagocytosis rate even if the concentration of LDL in the culture was as high as 1000 μg/ml (Fig. 4a, b).

### LDL enhanced the phagocytosis of GAS in human blood

**Fig. 5:**
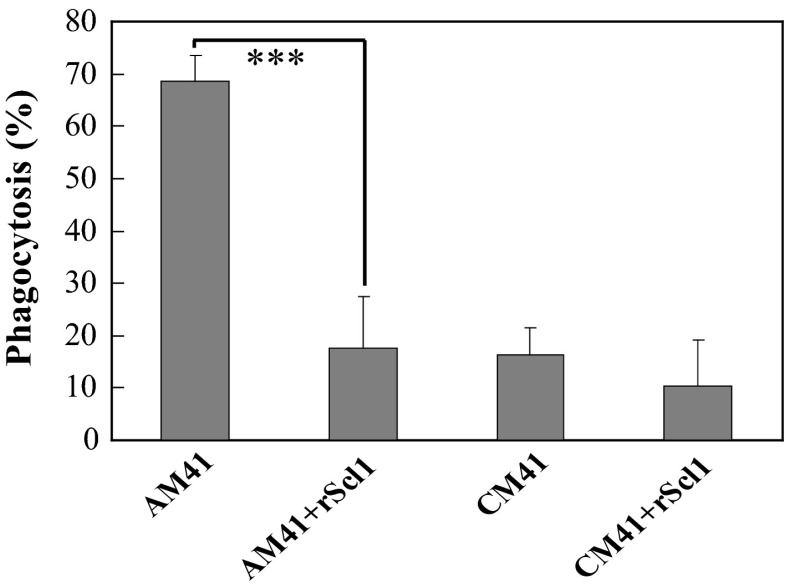
Phagocytosis of GAS in human blood. The 200 µl aliquot of fresh blood was pre-incubated with 50 µl rScl1 or PBS at 37 °C under agitation (170 rpm) for 1 min. A 100 µl aliquot of AM41-type or CM41-type GAS (~10^3^ cfu/ml) was added into the blood and agitated at 37 °C for 15 min. The colony-forming unit (cfu) was determined by plating samples on THY agar plates. The phagocytosis rates were expressed as 100 % × (cfu of addition − cfu after co-culture)/cfu of addition

To investigate whether LDL could promote the phagocytosis of GAS in human blood, we measured the viable number of AM41 and CM41-type GAS in whole blood with or without rScl1 after 15-min incubation. The phagocytic efficiency of AM41-type GAS was 69 % which was significantly higher than that of CM41-type GAS (16 %) (Fig. 5). The addition of rScl1 decreased the uptake of AM41-type GAS to 19 %, while phagocytosis rate for CM41-type GAS was reduced to 10 % (Fig. 5). Therefore, LDL also enhanced the killing of LDL-opsonized GAS in human blood.

## Discussion

GAS infection is an increasing problem, with >500,000 deaths per year [13]. Scl1–LDL interaction was found, but the significance of the interaction especially in the host immunity has not been uncovered. To the best of our knowledge, LDL was demonstrated for the first time to be an opsonin to enhance CD36-dependent opsonic phagocytosis of GAS by monocyte and whole human blood in this study.

In fact, LDL presents anti-*Klebsiella pneumoniae*, *K. pneumonia, Salmonella typhimurium, Listeria monocytogenes*, *Mycobacterium tuberculosis* and *Vibrio vulnificus* capability of preventing these bacterium infections [6]. LDL but not HDL binds to prions from the brains of patients with sporadic Creutzfeldt–Jakob disease to prevent the prion further invasion [14]; *Pseudomonas aeruginosa* was demonstrated to bind to oxLDL but not to LDL through protein PA0122 in a previous study [15] where the significance of the oxLDL–PA0122 interaction was not addressed. Our findings in this study demonstrated that the interaction of Sc11 and LDL played an important anti-infectious role. In fact, LDL may be an opsonin to enhance the phagocytosis of *Yersinia pestis* by monocyte due to the binding of LDL to *Y. pestis* [16]. However, pure pH6-Ag (similar to rScl1) can also competitively inhibit the binding of LDL to *Y. pestis* to abrogate LDL-mediated opsonophagocytosis of *Y. pestis*.

Oxidation of LDL may be a more important response of the host against infections; therefore, whether there exists an interaction of OxLDL with Sc11 and the implication of this interaction in the host innate immunity is worth pursuing.

We found for the first time that CD36 could be a much higher affinity receptor of the LDL opsonin receptor, because the results demonstrated that once LDL binds to GAS, LDL–GAS complex was quickly recognized by CD36 and phagocytosed by monocyte. The findings are not in line with the previous findings in other studies where scavenger receptors, including CD36, were previously thought to only mediate the non-opsonic phagocytosis of bacteria by monocyte/macrophage [17, 18].

Based upon our findings, monocyte or macrophage might ingest bacterium–LDL complex to protect against infections, which might result in the formation of foam cell, cholesterol-laden macrophage since LDL cholesterol is considered as a major factor in atherosclerosis development [19]. So far, the validity of the hypothesis that infection contributes to atherosclerosis has not been definitively established although the evidence is becoming compelling [20–22]. Our findings might shed a new light on the correlation between infections and atherosclerosis due to the double-edged sword property of LDL.

Severe invasive streptococcal infections are often associated with systemic dissemination, which reflects the diverse abilities of GAS to avoid eradication by phagocytic defenses. Therefore, GAS can be isolated from the bloodstream in most patients with invasive infection [23]. Polymorphonuclear neutrophils are the most important phagocytes in the blood; GAS, however, can avoid the killing by PMNs and survive within these cells [24]. Thus, LDL-mediated opsonophagocytosis of GAS by monocyte found in this study is particularly important for the rapid clearance of GAS by host since GAS inside the monocyte was killed within 1 h.

Our experiments were performed only in in vitro and ex vivo systems since GAS is a strictly human pathogen [25] and only a very few streptococcal strains can establish infection in mice. On the other hand, knockout of ApoB100 is lethal to animal. Therefore, a careful and profound strategy to further confirm the findings in this study in an in vivo system should be endeavored. Nevertheless, our findings that LDL was identified to be an opsonin to enhance CD36 mediated phagocytosis of GAS by monocyte and whole human blood may provide clues for anti-GAS infection and atherosclerosis prevention strategies.

## Electronic supplementary material

Supplementary material 1 (PDF 100 kb)

Supplementary material 2 (PDF 199 kb)

## Abbreviations

GAS: Group A *Streptococcus*
AM41: M41-type GAS (ATCC 12373)
CM41: M41-type GAS (CMCC 32198)
LDL: Low-density lipoprotein
