# Supplementary material for: LDL acts as an opsonin enhancing the phagocytosis of group A Streptococcus by monocyte and whole human blood
Source: Med Microbiol Immunol. 2015 Sep 21;205:155–62. doi: 10.1007/s00430-015-0436-8 (PMC4792331; doi:10.1007/s00430-015-0436-8)
Supplement: Supplementary file 1 — Supplementary material 1 (PDF 100 kb) [file 430_2015_436_MOESM1_ESM.pdf]

A

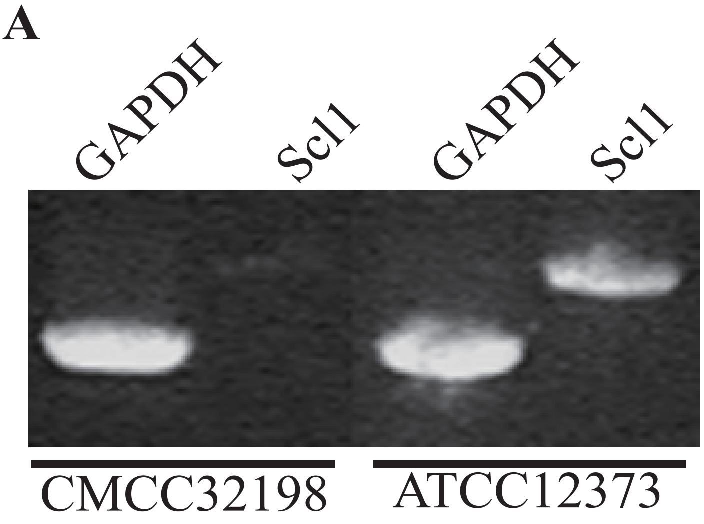

B

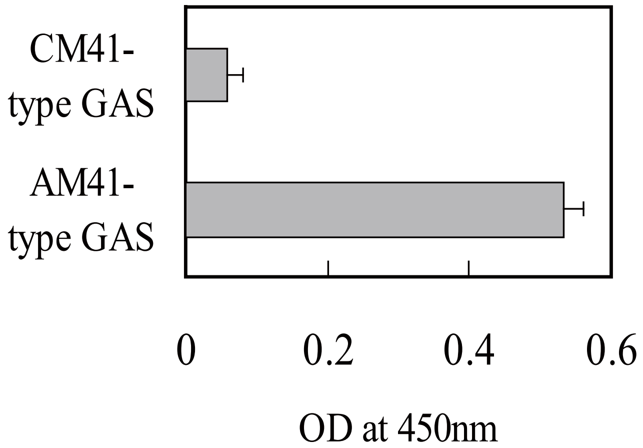

Figure S1. Expression of *Scl1* in GAS and its binding to LDL.
A. Quantification of mRNA encoding for Scl1 by RT-PCR. The mRNA encoding glyceraldehyde 3-phosphate dehydrogenase (GAPDH) was used as a reference. GAS cultures (10 ml) were grown in THY medium to mid-log phase (OD600 = ~0.5), and cell pellets were re-suspended in 500 μl of TE buffer [10 mMTris (pH 7.0), 1 mM EDTA] and treated with 30μl of mutanolysin (1 mg/ml) at 37°C for 5 min. 1ml of RNAiso Plus Total RNA extraction reagent (TaKaRa, Japan) was added into the above suspension and total RNA was extracted following the manufactory instruction. RNA-directed DNA synthesis was conducted with PrimeScriptTM II 1st strand cDNA Sythesis Kit (TaKaRa, Japan) using random hexamerprimers. We amplified scl1 using the following primers: forward primer (F), CTCCACAAAAGAGTGATCAGTC; reverse (R), TTAGTTGTTTTCTTTGCGTTT, and amplified glyceraldehyde-3-phosphate dehydrogenase (GAPDH) using the following specific primers: F, ATGGTAGGTCTCAGCGCTTGATTTTCATAAGGAGGAAATCACT; R, ATGGTAGGTCTCATATCATTTAGCAATTTTTGCGAAGTACTCA.
B. Binding assay between LDL and M41 Streptococcal cells. Plate was coated with cells (CM41 and AM41-type GAS), incubated with human LDL after washing three times with TBST (Tris-buffered saline supplemented with 0.05% Tween 20, pH 7.4). The bound LDL was detected with goat anti-LDL antibody (Sigma, USA) followed with HRP-conjugated donkey anti-goat (BD Biosciences) secondary antibody.
