# Supplementary material for: LDL acts as an opsonin enhancing the phagocytosis of group A Streptococcus by monocyte and whole human blood
Source: Med Microbiol Immunol. 2015 Sep 21;205:155–62. doi: 10.1007/s00430-015-0436-8 (PMC4792331; doi:10.1007/s00430-015-0436-8)
Supplement: Supplementary file 2 — Supplementary material 2 (PDF 199 kb) [file 430_2015_436_MOESM2_ESM.pdf]

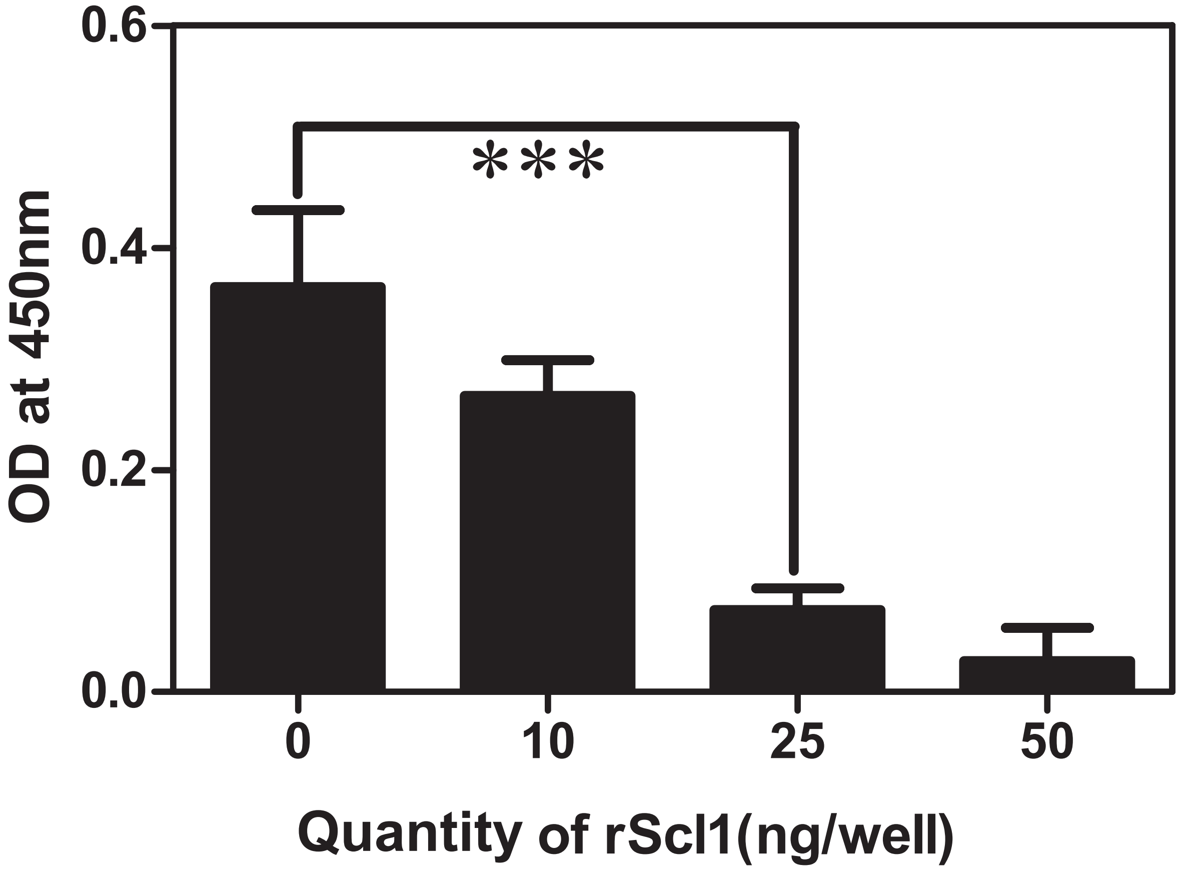

Figure S2. The interaction between LDL and AM41-type GAS was inhibited by rScl1.

The assay was conducted as described in Figure S1 except the addition of LDL containing rScl1 of 0, 10, 25 or 50 ng. 25 ng rScl1 is equivalent to 100 ng LDL in mole. The result indicated that rScl1 with concentration-dependent manner significantly inhibited the binding of LDL to AM41-type GAS. The binding capacity was decreased by 26.9%, 79.8% and 92.4% in the presence of rScl1 of 10, 25, 50 ng, respectively.
